# Discovery of QTL Alleles for Grain Shape in the Japan-MAGIC Rice Population Using Haplotype Information

**DOI:** 10.1534/g3.118.200558

**Published:** 2018-09-07

**Authors:** Daisuke Ogawa, Yasunori Nonoue, Hiroshi Tsunematsu, Noriko Kanno, Toshio Yamamoto, Jun-ichi Yonemaru

**Affiliations:** Institute of Crop Science, National Agricultural and Food Research Organization (NARO), Japan

**Keywords:** GWAS, haplotype, QTL, SNP, Multiparent Advanced Generation Inter-Cross (MAGIC), multiparental populations, MPP

## Abstract

A majority of traits are determined by multiple quantitative trait loci (QTL) that can have pleiotropic effects. A multi-parent advanced generation inter-cross (MAGIC) population is well suited for genetically analyzing the effects of multiple QTL on traits of interest because it contains a higher number of QTL alleles than a biparental population. We previously produced the JAPAN-MAGIC (JAM) population, derived from eight rice (*Oryza sativa* L.) cultivars with high yield and biomass in Japan, and developed the method of genome-wide association study (GWAS) using haplotype information on the JAM lines. This method was effective for identifying major genes such as *Waxy* for eating quality and *Sd1* for culm length. Here, we show that haplotype-based GWAS is also effective for the evaluation of multiple QTL with small effects on rice grain shape in the JAM lines. Although both the haplotype- and SNP-based GWAS identified multiple QTL for grain length and width, the sum of the estimated trait values of each allele for the QTL detected by haplotype-based GWAS had higher correlation with observed values than those detected by SNP-based GWAS, indicating high-accuracy QTL detection in the haplotype-based GWAS. Furthermore, the study revealed pleiotropic effects of some QTL regions in regulation of grain shape, suggesting that the haplotype-based GWAS using the JAM lines is an effective means to evaluate the main and side effects of haplotypes at each QTL. Information on the pleiotropic effects of haplotypes on various traits will be useful for designing ideal lines in a breeding program.

Rice is a major crop, especially in Asia, and is a staple food for billions of people in the world. Rice cultivars have been improved by breeding for thousands of years, today with the aid of DNA marker-assisted selection (Collard *et al.* 2008). To identify desirable genes for introduction, researchers have searched for DNA markers linked to the causative genes of agriculturally important traits, *e.g.*, *Waxy* for eating and cooking quality (Zhou *et al.* 2003), *SUB1A* for submergence tolerance (Xu *et al.* 2006), *Xa1* for bacterial leaf blight resistance (Yoshimura *et al.* 1998), and *pi21* for blast resistance (Fukuoka *et al.* 2009). These DNA markers have been developed through genetic analysis using biparental populations owing to their simple genetic structure.

Genome-wide association study (GWAS) is a powerful method to find QTL associated with target traits. Currently, GWAS is being performed using groups of cultivars and accessions as populations that are more genetically diverse than the biparental populations typically used in animal and plant breeding (Han and Huang 2013; Visscher *et al.* 2017). In rice, GWAS using a selected population consisting of 176 cultivars uncovered genes controlling agronomic traits such as days to heading and panicle length and number (Yano *et al.* 2016). However, GWAS is often problematic because complicated population structures hamper accurate detection of allelic effects on traits. One of the reasons for the success of Yano *et al.*’s study was careful selection of rice varieties that were not genetically highly structured or interrelated but had phenotypic diversity. Thus, there are bottlenecks restricting the elucidation of QTL alleles in GWAS when using genetically diverse populations, which we call “uncontrolled populations” in this paper.

Multi-parental inter-mated populations were first shown in mouse to enable analysis of higher numbers of genetic variations, which in turn give rise to wider phenotypic distributions (Churchill *et al.* 2004). In plants, the multi-parental inter-mated lines are generally referred to as multi-parent advanced generation inter-cross (MAGIC) lines. MAGIC lines have been developed in a number of plants including *Arabidopsis thaliana* (Kover *et al.* 2009), tomato (Pascual *et al.* 2015), faba (Sallam and Martsch 2015), cotton (Islam *et al.* 2016), wheat (Huang *et al.* 2012; Mackay *et al.* 2014), barley (Sannemann *et al.* 2015), maize (Dell’Acqua *et al.* 2015), sorghum (Ongom and Ejeta 2018), and rice (Bandillo *et al.* 2013; Meng *et al.* 2016a; Meng *et al.* 2016b; Ogawa *et al.* 2018). MAGIC populations are intermediate between biparental populations and uncontrolled populations in terms of genetic complexity and diversity. The population structure of MAGIC lines is not as complicated as that of uncontrolled populations because there are fewer alleles and recombination events. Genetic analyses of the MAGIC populations require a high density of DNA polymorphisms to distinguish chromosomal segments with low linkage disequilibrium derived from multiple parents, but recent progress in DNA sequencing technology facilitates the identification of high numbers of SNPs and insertion/deletion mutations. A MAGIC population derived from 19 *Arabidopsis thaliana* accessions was analyzed at the level of haplotype, which is defined as a chromosomal segment from a founder (Kover *et al.* 2009). The program for haplotype estimation is freely available. These tools facilitate GWAS using newly developed MAGIC populations to examine genetic variations for traits of interest.

We produced the JAPAN-MAGIC (JAM) population by using four *japonica* cultivars and four *indica* cultivars that have high grain yield and biomass when cultivated in Japan (Ogawa *et al.* 2018). In the process of developing the JAM population using the single-seed descent (SSD) method, lines were randomly selected from among those that produced any amount of seed. Sterility caused by hybridization between *japonica* and *indica* cultivars can lead to distortion of the founder genome proportions in the resulting population, but in this study the proportions of haplotypes among the eight founders were almost equal, at least at the whole-genome level, to their proportions in the JAM lines (Ogawa *et al.* 2018). This finding indicates that the JAM lines are suitable for evaluation of alleles from the eight founders. The JAM population shows higher phenotypic diversity than a set of four biparental recombinant inbred lines (RILs) produced from the same eight parents used to generate the JAM lines. GWAS using haplotype data enabled us to determine precise positions of QTL and to discriminate multiple alleles for major genes such as *Waxy* and *Sd1* (Ogawa *et al.* 2018). This result indicated that haplotype-based GWAS can be used to detect genes with major effects. However, it was still unknown whether haplotype-based GWAS was effective for analyzing traits controlled by multiple factors.

Genetic studies of QTL using various populations mainly focus on effects of QTL on expression of specific target traits, but QTL often have pleiotropic effects. In rice, *qPE9-1*, detected as a QTL for panicle erectness, also affects plant height and panicle length (Yan *et al.* 2007). *GHD8/DTH8*, detected as a QTL for heading date, affects the CO_2_ assimilation rate in photosynthesis, plant height, and grain yield (Adachi *et al.* 2017; Yan *et al.* 2011). To produce ideal lines through DNA marker-assisted selection using QTL information, we have to know not only the main effects of QTL for target traits but also their side effects, which may be detrimental to crop performance. From this aspect, the pleiotropic effects of QTL should be paid more attention in molecular breeding.

In this study, we applied the haplotype-based GWAS method to the JAM lines to identify QTL associated with grain shape, which is known to be regulated by many genetic factors (Li *et al.* 2018; Nagata *et al.* 2015; Zuo and Li 2014). By comparing this method with ordinary SNP-based GWAS, we validated the accuracy of the haplotype-based GWAS method. Furthermore, we focused on the pleiotropic effects (both main and side effects) of the QTL for grain shape, which were detected in the haplotype-based GWAS using the JAM lines. This analysis shed light on the different effects of the QTL in the regulation of grain shape.

## Materials and Methods

### Development of the JAM population

As described in a previous paper (Ogawa *et al.* 2018), the JAM population was developed from eight founders: Ruriaoba (RU), Hokuriku 193 (HO), Takanari (TK), Suweon 258 (SU), Mizuhochikara (MI), Bekogonomi (BE), Tachiaoba (TC), and Akidawara (AK). On the basis of variant analysis of all annotated genes using SnpEff software (Cingolani *et al.* 2012), the first four listed cultivars were identified as *indica* and the second four as *japonica* (Ogawa *et al.* 2018). This result is consistent with a phylogenetic analysis of 126 rice accessions, including five of the founders of the JAM population, which was performed using 1046 genome-wide SNP markers in our previous paper (Yonemaru *et al.* 2014). We first crossed each *indica* cultivar with a different *japonica* cultivar to produce four types of hybrid seed. These hybrids were crossed to produce four-way recombinants and then crossed again to produce eight-way recombinants. We originally planned to produce 1,000 JAM lines (F_2_) by developing 10 lines from each of 100 eight-way recombinants. In fact, we obtained F_6_ seeds from 981 JAM lines through the SSD method and used 372 JAM lines (F_5_) for this study.

### Cultivation of JAM lines

Germinated seeds were sown in trays filled with soil on May 9, 2016 and incubated at 30° in the dark for 2 days. Seedlings were grown in a paddy field (Kannondai District, Tsukuba, Japan) covered with thin plastic film to protect the plants from the cold. Individual seedlings were transferred to another paddy field on June 8, 2016 and cultivated according to standard procedures at the National Agricultural Research Organization (NARO) in Tsukuba.

### Analysis of grain shape

To collect rice grains from panicles, the panicle part containing the grains was put into a glass Petri dish, with the panicle base remaining outside of the dish. The grains were removed by using the lid of the Petri dish to compress the panicle against the bottom edge of the dish and pulling the panicle base away from the dish, thus leaving the grains inside the dish. Awns and pedicels were removed by rubbing the grains in a bag and then by vacuum. Any awns and pedicels that remained were cut off with scissors. We put grains of each JAM line directly on the glass of a scanner (Seiko Epson, Suwa City, Japan) and aligned them to avoid contact between adjacent grains. We scanned the grains (600 dpi) and analyzed the images (0.042433 mm/pixel) using the SmartGrain software program (Tanabata *et al.* 2012), which can automatically detect every single grain in an image and evaluate its length, width, and area. The number of grains per plant ranged from 11 to 628 in the 372 JAM lines. The averages of grain length, width, and area of the JAM lines were used for GWAS.

### GWAS and Sum of the Additive effects of QTL (SAQ)

SNP and haplotype data for the JAM lines were obtained in our previous work (Ogawa *et al.* 2018). A total of 16,345 SNP locations were detected by GBS analysis of the 376 JAM lines. The haplotype data for the JAM lines were predicted by using the method proposed for haplotype prediction in an Arabidopsis MAGIC population (Kover *et al.* 2009). All parameters were set to their defaults, except “-p”, which was set to 2.

SNP-based GWAS was performed using R software (https://www.r-project.org/). Associations of the imputed genotype data with phenotype data for grain length and width were analyzed with the General Linear Model (GLM) and Mixed Linear Model (MLM). The GLM was applied for the naïve (population structure not considered) and Q (population structure results included as fixed effects) analyses. Population structure was determined by principal component analysis (PCA) using the R function “prcomp”. The number of principal components (PCs) was set as 6 because the variance of PC score decreased gradually thereafter (Figure S1). The K (kinship results included as fixed effects) and QK models were analyzed with MLM (Yu *et al.* 2006) implemented in the “GWAS” function of the “rrBLUP” package (Endelman 2011). The kinship data were computed by using the “A.mat” function of “rrBLUP.”

Haplotype-based GWAS was performed by using haplotype information at each of the SNPs. To distinguish the newly identified QTL from previously identified QTL for rice grain shape, we named the newly identified QTL as *JAM-GL* for grain length and as *JAM-GW* for grain width. Both *JAM-GL* and *JAM-GW*, which were identified by using haplotype-based GWAS, were analyzed with non-parametric ANOVA (Kruskal–Wallis rank sum test) using the R function “kruskal.test”.

Peaks of the 5, 10, 20, and 30 highest −log_10_ P values in the results of both SNP-based and haplotype-based GWAS analyses were detected using the “findpeaks” function (minpeakdistance = 100) in the “pracma” package, and the SNPs associated with the peaks were defined as the QTL. Estimates of the additive effects of these QTL were calculated by the means of grain length and width for each SNP allele or haplotype using the R function “tapply”, and the sum of the additive effects of QTL (SAQ) was calculated.

Scatter plots were generated using the “ggplot2” package in the R software. These plots were used to show correlations between SAQ and observed values, and to illustrate the effects of haplotype at each QTL position on grain length and width.

### Sequences of GW2 alleles

The sequences of *GW2* alleles in the founders were determined using short-read Illumina resequencing data (Ogawa *et al.* 2018). The mutations in these alleles were analyzed for their effects on gene function in SnpEff software (Cingolani *et al.* 2012), which provides the number of variations that belong to one of four categories (variants_impact_high, _moderate, _low, or _modifier) according to their putative effect. Allele classification was performed as reported previously (Ogawa *et al.* 2018).

### Data availability

Germplasm of JAM lines and founders, and their haplotypes and phenotypes, are available by request. They are offered through a material transfer agreement (MTA) from NARO via Daisuke.Ogawa@affrc.go.jp. Supplemental material available at Figshare: https://doi.org/10.25387/g3.7027991.

## Results

### The JAM lines have phenotypic diversity for grain shape

**Figure 1:**
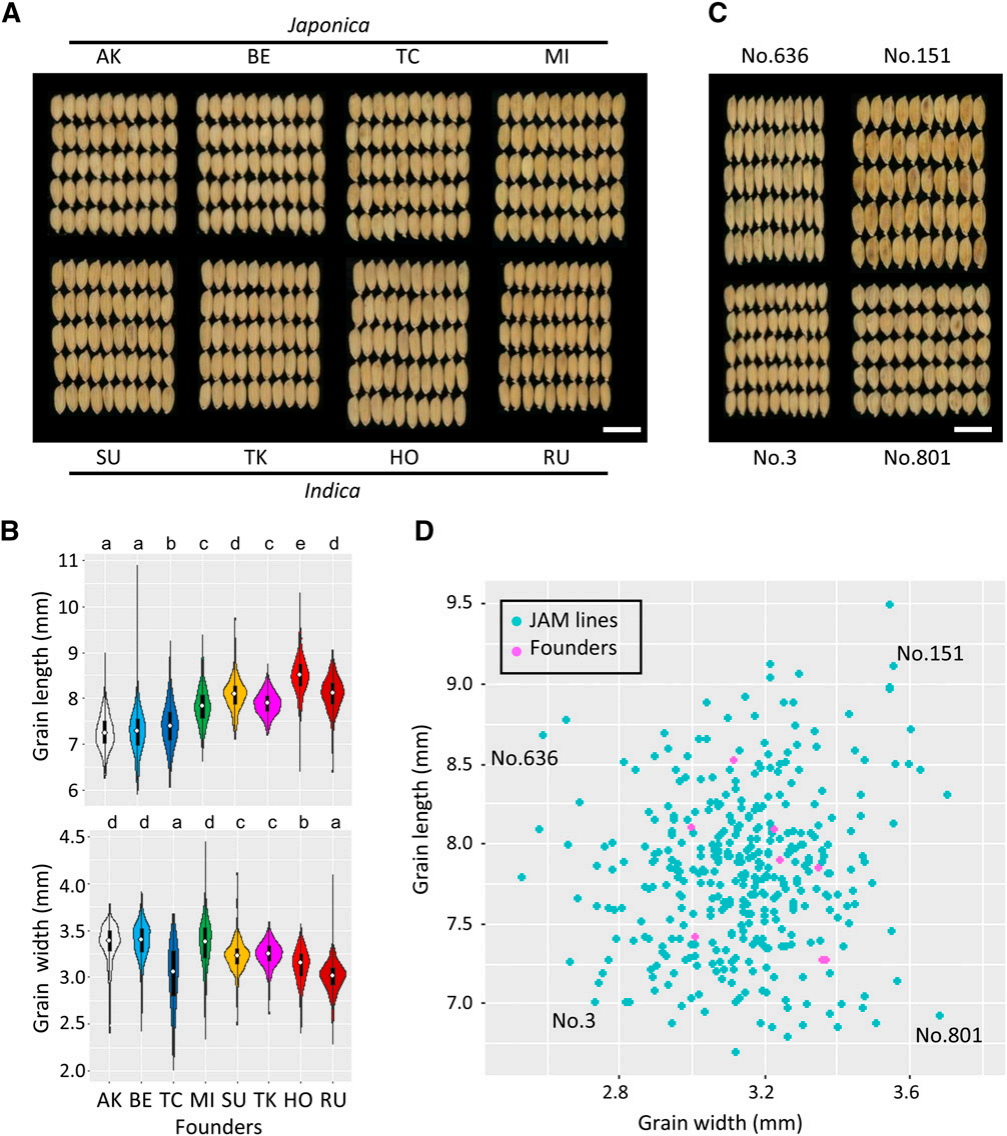
Grain length and width of the eight founders and JAM lines. (A) Alignments of 50 grains of each of the four *japonica* cultivars (Akidawara [AK], Bekogonomi [BE], Tachiaoba [TC], and Mizuhochikara [MI]) and four *indica* cultivars (Suweon 258 [SU], Takanari [TK], Hokuriku 193 [HO], and Ruriaoba [RU]) used as founders. Pictures of the grain of each founder were taken individually and then merged. (B) Violin plots of the grain length and width of the founders. They include information on kernel density estimation, quantiles (black boxes), and median values (white circles). Different letters indicate significant differences (Tukey method, *P* < 0.05). (C) Alignments of 50 grains each of four JAM lines showing characteristic features. (D) Scatter plot of average grain length and width for each JAM line and the eight founders. White bars indicate 1 cm.

The eight founders of the JAM lines include four *japonica* cultivars and four *indica* cultivars. The grain of *japonica* cultivars is generally more round and that of *indica* more slender (Misra *et al.* 2017; Nagata *et al.* 2015). This trend could be seen in the founders: for example, the grain shape of *japonica* founders AK and BE was significantly shorter and wider than that of the *indica* founders, whereas the grain of *indica* founders HO, SU, and RU was significantly longer and narrower than all but one of the *japonica* founders (Figure 1A and B).

To understand the phenotypic distribution of grain shape in the 372 JAM lines, we evaluated their grain length and width (Figure 1C and D). The grain length ranged from 6.7 to 9.5 mm and grain width from 2.5 to 3.7 mm. The distributions of both grain length and grain width were continuous, and the two characteristics were not obviously correlated. Compared to the eight founders, JAM lines with longer, shorter, narrower, and wider grain were found, indicating transgressive segregation. This result is consistent with the involvement of multiple genetic factors in grain shape. We also checked the grain areas of the JAM lines. Grain area was highly correlated with both grain length and grain width (Figure S2), suggesting that grain length and width contribute to grain area almost equally.

### Haplotype-based GWAS using the JAM lines is useful for detection of QTL for grain shape

**Figure 2:**
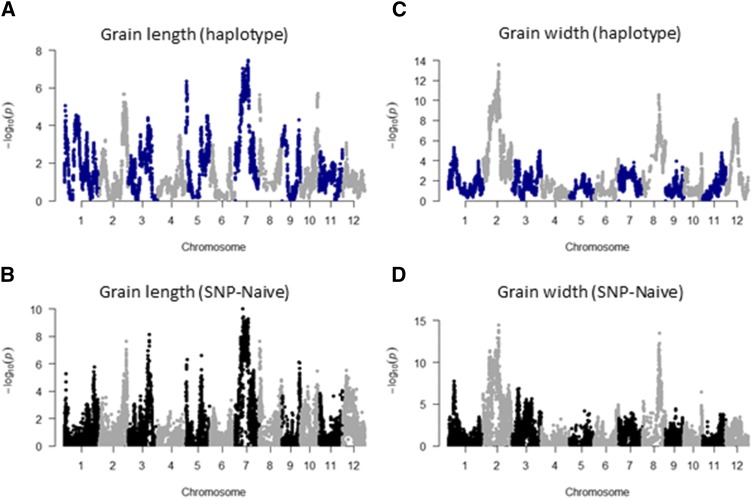
Manhattan plots of GWAS on grain length and width measurements. GWAS was carried out for (A, B) grain length and (C, D) width using (A, C) haplotype data and (B, D) SNP data. The naïve model was used for the SNP-based GWAS.

To clarify what factors regulate grain shape, we carried out haplotype-based GWAS and SNP-based GWAS with a naïve model (Figures 2 and S3). Both analyses identified several QTL for which the −log_10_ P value was more than 5, indicating that grain shape is a complex trait regulated by several factors. We also performed SNP-based GWAS with a Q model for considering population structure, K model for considering kinship, and QK model for considering both (Figure S4). The plot pattern of the SNP-based GWAS with the Q, K, and QK models was substantially different from that of SNP-based GWAS with the naïve model and the haplotype-based GWAS, confirming that the result of GWAS is changed by consideration of the population structure and kinship. However, what method is proper for the GWAS to detect QTL for the trait of grain shape is unknown.

To answer the question, we investigated the accuracy of QTL detection by checking the correlation between SAQ on grain length and width and the actual values in each JAM line. We determined the SNP positions of QTL using the software program “findpeaks” and chose the top 5, 10, 20, and 30 QTL positions (Tables S1 and S2). For both traits, the correlations between QTL additive effects and observed values in haplotype-based GWAS tended to be higher than those for SNP-based GWAS with the naïve, Q, K, and QK models (Tables 1 and 2). Especially for the top 5 QTL, the pattern of correlation in the haplotype-based GWAS was remarkably different from the others, *i.e.*, the data points were aligned less vertically (Figures S5 and S6). That might be attributable to the difference in the number of possibilities between haplotype-based and SNP-based GWAS: for 5 QTL, the former is 32,768 (8^5^) because there are 8 possible haplotypes per locus and the latter is 32 (2^5^) because there are 2 possible SNP alleles per locus. These results suggest that haplotype-based GWAS accurately detects the positions of QTL for grain shape.

**Table 1:** Pearson correlations between the observed values of grain length in the JAM lines and the sum of the additive effects of QTL (SAQ) obtained from the five models tested

Number of QTL	Haplotype-based model	Naïve model	Q model	K model	QK model
5	0.53	0.46	0.39	0.44	0.45
10	0.60	0.61	0.49	0.59	0.54
20	0.71	0.66	0.62	0.70	0.65
30	0.76	0.69	0.72	0.75	0.74

All of the correlation values in the five models are significant (*P* < 0.001).

**Table 2:** Pearson correlations between the observed values of grain width in the JAM lines and the sum of the additive effects of QTL (SAQ) obtained from the five models tested

Number of QTL	Haplotype-based model	Naïve model	Q model	K model	QK model
5	0.56	0.49	0.50	0.55	0.47
10	0.66	0.58	0.60	0.63	0.50
20	0.72	0.68	0.65	0.72	0.67
30	0.76	0.70	0.70	0.76	0.73

All of the correlation values in the five models are significant (*P* < 0.001).

### The haplotype-based GWAS of grain length and width uncovered the main and side effects of the QTL

**Figure 3:**
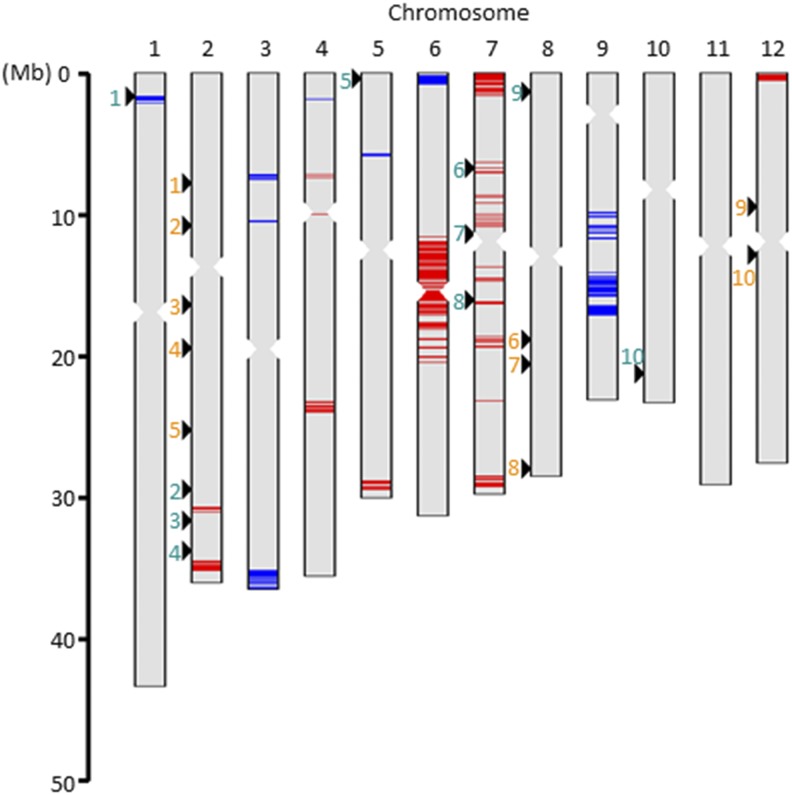
Chromosomal positions of QTL and haplotypes with effects on grain length and width. Arrowheads indicate the QTL positions of *JAM-GL1 to -GL10* (cyan) and *JAM-GW1 to -GW10* (orange). Horizontal lines on the chromosomes indicate regions where haplotypes have correlated effects on grain length and width. Red and blue indicate positive and negative Pearson correlations (*P* < 0.05), respectively.

Genetic studies generally focus on the main effects of QTL (*i.e.*, those affecting only the target trait) but not their influence on other traits (here called side effects), despite the known existence of pleiotropic effects of QTL. We asked whether QTL for grain length affect grain width and vice versa. We plotted averages of grain length and width in haplotypes at the positions of the top 10 QTL for grain length and width detected in the haplotype-based GWAS (designated *JAM-GL1 to -GL10* and *JAM-GW1 to -GW10*; Figures S3, S7, and S8; Tables S3 and S4). In addition, we calculated the Pearson correlation values between grain length and width for the entire set of SNPs (Figure 3). This analysis revealed both negative and positive correlations between grain length and width among haplotype effects at the position of *JAM-GL1* and in the regions of *JAM-GL6 to -GL8*, respectively. In other words, some genome positions with major QTL affecting one grain shape trait also had effects on the other trait. This result implies that haplotype-based GWAS using the JAM lines has the ability to reveal haplotype effects both on the target trait and on other traits at the location of each QTL.

### The regions of JAM-GLs and JAM-GWs identified by haplotype-based GWAS partially overlapped those of previously detected QTL

We examined whether the QTL identified in this study overlapped with those previously detected in a genetic study of grain shape using biparental RILs of *japonica* and *indica* cultivars (Nagata *et al.* 2015). A total of 6 out of the top 20 QTL identified in the present study overlapped with those previously detected by Nagata *et al.* (Table S5). In that study, QTL were detected in a BC_4_F_2_ population produced by crosses between *japonica* cultivar Koshihikari and *indica* cultivar IR-64 (Nagata *et al.* 2015). This finding indicates that these QTL may be positioned as common QTL for variation of grain shape in *japonica* and *indica*.

We searched for candidate genes for the QTL by using haplotype data and sequence information for the eight founders. Intriguingly, most of the QTL detected in this study did not seem to correspond to the previously identified genes listed in Table S6 (Li *et al.* 2018; Zuo and Li 2014): either the allelic pattern was inconsistent with the effect of haplotype on grain shape or the QTL were located more than 1 Mbp away from the position of known grain shape regulators. Among known genes, only *GW2* is located near a QTL identified in this study, *JAM-GW1*. Analysis of resequencing data (Ogawa *et al.* 2018) of *GW2* alleles of the founders revealed 26 mutations including SNPs and 1-bp insertion/deletion mutations (Figure S9). Four *GW2* alleles were found in the eight founders but mutations changing amino acid sequence of GW2 and detected as functional SNPs (FNPs) in a previous report (Song *et al.* 2007) did not exist in the JAM founders.

## Discussion

Here, we showed that haplotype-based GWAS of the JAM lines produced higher correlation values between “sum of the additive haplotype effects of QTLs on the grain shape” and “actual values of grain shape” than did ordinary SNP-based GWAS with various models. This indicates that the peaks in the haplotype-based analysis can accurately detect QTL for grain shape, which is controlled by multiple QTL with small effects. These results suggest that haplotype-based GWAS using the JAM lines is effective for detecting QTL associated with complex traits.

Although GWAS using MAGIC and uncontrolled populations have revealed effective alleles for various traits, the ordinary method of GWAS based on biallelic SNPs often results in inconsistency between SNP alleles and functional alleles because of low coverage of SNPs and multi-alleles, often leading to imprecise detection of QTL. In this study, we showed that genome-wide haplotype information on the JAM lines overcomes this problem, as illustrated by the clearer peaks in the Manhattan plots. As suggested by our findings with the JAM lines, haplotype information rather than biallelic SNP information could be also useful in GWAS of uncontrolled populations, although the difficulty of haplotype estimation depends on the population. Recently, the ever-increasing availability of rich genome sequence information and improved algorithms for haplotype prediction have increased the accuracy of genetic analyses. In cassava, the haplotype map identified a deleterious mutation that concomitantly altered starch and ketone metabolism pathways during domestication (Ramu *et al.* 2017). In sweet potato, a haplotype-based analysis uncovered the evolutional history of chromosomal regions and polyploidy (Yang *et al.* 2017). Thus, genetic approaches using haplotype information are becoming standard for the detection of QTL.

The haplotype-based GWAS of grain length and width using the JAM lines revealed both the main and side effects of haplotypes on grain shape at each QTL. This demonstrates the importance of genetic analysis to understand trait–trait interaction because the scatter plots of grain length and width in the JAM lines did not show significant positive or negative relationships between these traits. Grain shape is regulated through signaling pathways such as G proteins, proteasomes, phytohormones, and MAP kinases (Li *et al.* 2018; Zuo and Li 2014). These signaling molecules affect grain shape in different ways; as shown in Table S6, some genes affect either length or width independently, whereas others affect grain area or length/width ratio. From this knowledge, it is understandable that the JAM lines contain genomic regions exhibiting haplotype effects on more than one grain shape trait. Although studies of trait–trait interactions are possible using biparental populations, MAGIC populations may be more advantageous because of the higher number of haplotypes. The haplotype-based GWAS method for genetic analysis using JAM lines should also be applicable to traits other than grain shape.

In summary, our results showed that the haplotype-based GWAS in the JAM lines was effective for detecting QTL associated with grain shape, which is an important trait for grain yield. The haplotype-based GWAS method can provide information on both main and side effects of QTL for agronomic traits, and the obtained information will contribute to producing ideal lines.
